# A Rare Case of Fungal Keratitis Caused by Plectosphaerella cucumerina Diagnosed With Repeated Corneal Scrapings: A Case Report

**DOI:** 10.7759/cureus.27628

**Published:** 2022-08-03

**Authors:** Takuya Kiriyama, Takehiro Hariya, Masaaki Yoshida, Daisuke Todokoro, Toru Nakazawa

**Affiliations:** 1 Department of Ophthalmology, Tohoku University Graduate School of Medicine, Sendai, JPN; 2 Department of Ophthalmology, Gunma University, Graduate School of Medicine, Maebashi, JPN

**Keywords:** repeated corneal scraping, voriconazole, plectosphaerella cucumerina, fungal keratitis, infectious keratitis

## Abstract

*Plectosphaerella cucumerina* is a filamentous fungus that infects plants and crops, but there are few previous reports of human infections. The current case was an 82-year-old woman who was referred to us for corneal infection in her left eye that did not improve with antibacterial and anti-inflammatory treatment. The best-corrected visual acuity (BCVA) of the eye at the first visit to us was hand motion. Slit-lamp examination revealed extensive white infiltration and ulceration in the anterior corneal stroma of the left eye. Intensive antibacterial and antiviral treatment for one month did not improve the condition of the cornea. Although initial culture testing and polymerase chain reaction (PCR) testing of corneal scraping samples did not reveal the causative microorganism, repeated culture testing identified *P. cucumerina.* The corneal infection eventually subsided after topical and systemic treatment with voriconazole (VRCZ). The final BCVA in the left eye was 1.3 logMAR. This was a rare case of fungal keratitis due to *P. cucumerina.* Our case suggests that it is important to perform repeated examinations with corneal scrapings, especially when the treatment response is poor.

## Introduction

Fungal keratitis, especially filamentous fungal keratitis, typically occurs in patients with a history of ocular trauma associated with plants [1]. Thus, agricultural workers are at a higher risk of fungal keratitis [2]. *Plectosphaerella cucumerina* is a filamentous fungus that infects plants and crops. However, there have been only two reported cases of human keratitis infection in which the causative organism was identified with DNA sequencing or matrix-assisted laser desorption ionization-time of flight mass spectrometry (MALDI-TOF MS) [3,4].

Diagnosing infectious keratitis, including fungal keratitis, is sometimes difficult because the very small size of corneal scraping samples results in culture testing often returning false negatives. This report presents a case of keratitis caused by *P. cucumerina* that was successfully diagnosed with repeated corneal-scraping culture testing; this testing contributed to choosing an appropriate treatment strategy.

## Case presentation

Case report

**Figure 1:**
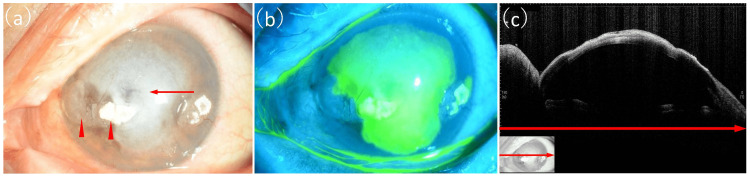
Corneal findings from the left eye at the first visit. (a) Slit-lamp photograph showing band-shaped keratopathy (indicated by red arrowheads), extensive white infiltration (indicated by the red arrow) covering more than two-thirds of the entire cornea, larger epithelial defects, and ulcer extending into the anterior corneal stroma with mild conjunctival hyperemia. (b) Fluorescein-staining photograph showing an extensive corneal ulcer. (c) Anterior segment optical coherence tomography image showing high reflectivity from the corneal epithelium to the shallow layer of the corneal stroma. The red arrows show corresponding areas.

An 82-year-old woman was referred to us for a corneal infection in her left eye that did not improve with 0.5% levofloxacin eyedrops (4 times/day) and 0.1% fluorometholone eyedrops (4 times/day) for two weeks. The best-corrected visual acuity (BCVA) of the eye at the first visit to us was hand motion, while that of the fellow eye was −0.1 logMAR. A slit-lamp examination revealed band-shaped keratopathy in both eyes. Extensive white infiltration covered more than two-thirds of the entire cornea, with larger epithelial defects and ulcers extending into the anterior corneal stroma of the left eye, in addition to mild conjunctival hyperemia (Figure 1).

**Figure 2:**
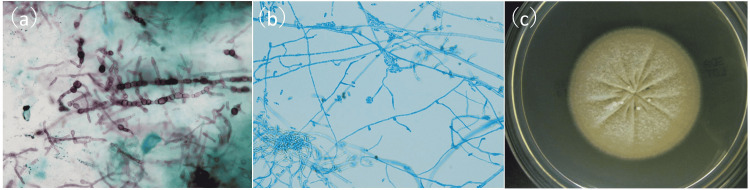
Findings from culture testing of corneal scrapings. (a) Smear of the corneal scrapings with Grocott’s stain (×800) showing filamentous fungi in the form of hyphae with septa (shown by black dye). (b) Smear of a colony cultured from the corneal scrapings with lactophenol cotton blue stain (×400). The colony is spindle-shaped with rounded and elongated ends; one end is slightly more curved. (c) Culturing with Sabouraud agar medium at room temperature for 10 days showed a light cream color.

The patient had a history of ocular trauma: her left eye had been hit with an umbrella about four months before the onset of symptoms. We suspected infectious keratitis and obtained corneal scrapings for culture testing and polymerase chain reaction (PCR) testing. We examined the samples for human herpesvirus 1 to 8, bacterial 16S rRNA, and fungal 28S rRNA. We started treatment with 0.5% moxifloxacin eye drops every hour, 0.5% arbekacin eyedrops every two hours, 3% acyclovir eye ointment five times a day, 1% atropine eyedrops once a day, oral minocycline (400 mg/day), and valacyclovir (3000 mg/day). *Staphylococcus epidermidis* was detected in the first round of culture testing, but examination with a microscope did not reveal phagocytosis of this bacterium. PCR testing of the same corneal scraping sample for herpesvirus, bacterial 16S rRNA, and fungal 28S rRNA was negative. Nevertheless, the corneal findings did not improve even after one month of intensive antibacterial and antiviral treatment. Thus, we obtained a new corneal scraping sample and repeated the culture testing; we also started antifungal treatment with 1.0% voriconazole (VRCZ) eyedrops, as we suspected fungal keratitis. In the second round of culture testing, *Enterobacter sakazakii* was detected, but this was considered to be a transient organism. We performed the third round of culture testing a month later due to the poor treatment response and detected filamentous fungi in culture testing and examination with a microscope. Findings related to the fungus are shown in Figure 2.

We increased the frequency of the VRCZ eyedrops to every hour and added oral VRCZ. DNA sequencing from the cultured colony identified *P. cucumerina*, as described in the section below. Susceptibility testing was performed according to the CLSI M38-A2 standard. The results revealed a low minimum inhibitory concentration (MIC) for VRCZ (1 µg/mL), as shown in Table 1, so we continued treatment with VRCZ.

**Table 1:** Susceptibility test of antifungal agents.

Antifungal agents	Minimum inhibitory concentration (μg/ml)
Micafungin	1
Caspofungin	2
Amphotericin B	2
Pimaricin	2
5-Fluorocytosine	>64
Fluconazole	16
Itraconazole	1
Voriconazole	1
Miconazole	1

**Figure 3:**
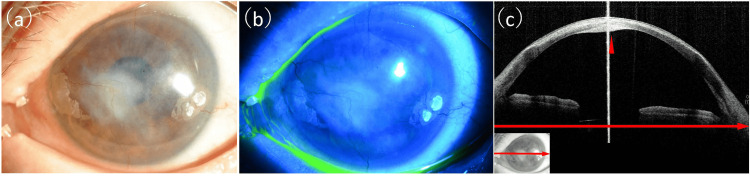
Corneal findings from the left eye after four months of antifungal treatment. (a) Slit-lamp photograph showing that the corneal infection had subsided. Corneal scarring remained and corneal neovascularization occurred. (b) Fluorescein-staining photograph showing the healed corneal epithelium. (c) Anterior segment optical coherence tomography image showing recovery of the corneal structure. High reflectivity in the shallow layer of the stroma and thick deposits (indicated by the red arrowhead) under the corneal endothelium remained. The red arrows show corresponding areas.

After four months of antifungal treatment, the corneal infection eventually subsided, but corneal scarring remained (Figure 3).

**Figure 4:**
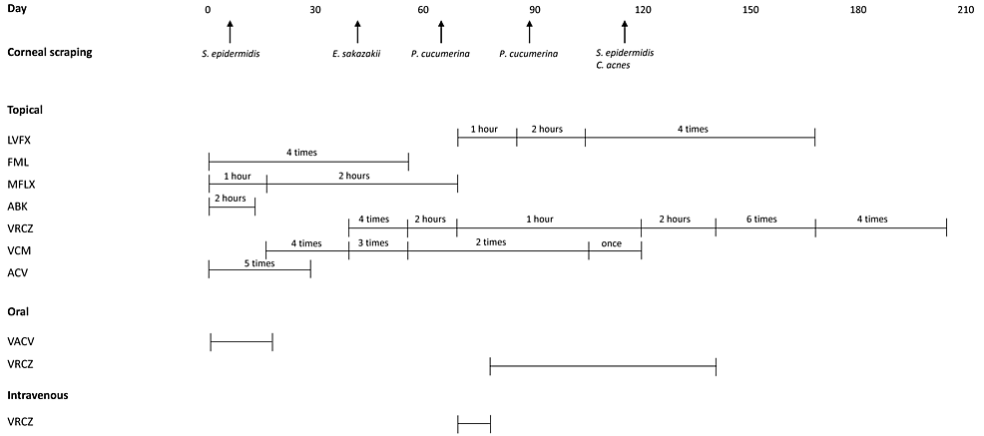
The drugs used for treatment and the results of corneal scraping culture testing. LVFX: levofloxacin, FML: fluorometholone, MFLX: moxifloxacin, ABK: arbekacin, VRCZ: voriconazole, VCM: vancomycin, ACV: aciclovir, VACV: valaciclovir, MINO: minocycline, X times: X times/day, X hours: every X hours, *S. epidermidis:* *Staphylococcus epidermidis*, *E. sakazakii*: *Enterobacter sakazakii*, *P. cucumerina:* *Plectosphaerella cucumerina*, *C. acnes:* *Cutibacterium acnes.*

The final BCVA in the left eye was 0.05. The drugs used in the treatment are shown in Figure 4.

Identification of fungus

We performed PCR amplification of the ITSrDNA and 28SrDNA (D1/D2) and β-tubulin 2 partial sequences of the isolated fungal strain (LSEM3737). All base sequences showed 100% homology with *P. cucumerina* according to data obtained from Mycobank Pairwise sequence alignment and a BLAST search against the DDBJ/EMBL/GenBank of the DNA data bank. The deposited GenBank accession numbers of *P. cucumerina* were MN915127 for ITSrDNA, LR590376 for 28SrDNA (D1/D2), and MG029465 for β-tubulin 2.

## Discussion

*P. cucumerina* is a filamentous fungus that inhabits rhizosphere soils and infects potato, tomato, and radish roots. It has been isolated from various parts of the world [5]. The current patient was not an agricultural worker. Thus, the route of infection was not clear, although the patient had a history of ocular trauma caused by being hit with an umbrella four months prior to the onset of keratitis. Previously, only two cases of human keratitis infection have been reported [3,4]. Both cases had contact with plants or soil. One was a case of keratitis caused by *P. cucumerina* (a synonym for *P. tabacinum*) in a 78-year-old male patient with no history of ocular trauma or trauma associated with plants, and the other was a 74-year-old male who rubbed his left eye after he finished farm work. Drug susceptibility testing in these cases revealed a high susceptibility to VRCZ but not to fluconazole (FLCZ) or miconazole (MCZ). The main treatment in both cases was with VRCZ, similar to our patient. In our case, the MIC for FLCZ was high, but the MIC for MCZ was not. Thus, generally, treatment with VRCZ may be a good option for fungal keratitis caused by *P. cucumerina*. Nonetheless, the visual outcome was poor in both the previously reported case and the current case; the final affected-eye BCVA was 0.02 and 0.05, respectively.

Repeated culture testing successfully diagnosed the current case of fungal keratitis, even after no fungi were detected in the first and second rounds of smear examination and culture testing. In a multicenter, prospective, observational study of fungal keratitis in Japan, the causative fungal organism was isolated in only 71 of 133 cases [6]. Our experience, in this case, suggests that repeated examination can increase the chance of successfully detecting the causative microorganism. Although PCR testing for fungal 18S rRNA, 28S rRNA, and ITS has previously been used to attempt to diagnose fungal keratitis [7-9], the current case did not have a positive result for fungal 28S rRNA in the first round of testing of corneal scraping samples. Thus, culture testing might still be considered the gold standard for diagnosing fungal keratitis. Culture testing also has other advantages: it can be used to identify the species of the causative microorganism, and the cultured microorganisms can be used to perform susceptibility testing. However, it is sometimes difficult to identify fungal species based only on morphological evaluation. For example, the Fusarium solani species complex is a representative species in fungal keratitis with a morphology similar to that of *P. cucumerina*. Pathologically, *P. cucumerina* is characterized by a spindle shape with rounded and elongated ends; one end is slightly more curved, in contrast with the Fusarium species complex, which is characterized by "boat-shaped" macroconidia [10]. These characteristics are sometimes hard to detect in smear examinations of corneal scrapings, as shown in Figure 2(a), potentially resulting in cases of *P. cucumerina* keratitis being misdiagnosed as Fusarium keratitis. In addition to pathological evaluation with cultured microorganisms, performing DNA sequencing or MALDI-TOF MS should be beneficial for identifying the causative microorganism, although it might be hard to perform this testing as part of routine care due to the specialized knowledge and equipment that are required.

## Conclusions

We observed a rare case of fungal keratitis caused by *P. cucumerina*. The visual prognosis was poor, but an accurate diagnosis, enabled by repeated culture testing of corneal scraping samples and DNA sequencing, contributed to the selection of the most appropriate treatment. Our findings show that it is important to identify the causative organism in such cases by performing repeated examinations, even if the first examination is negative.

## References

[1] Hoffman JJ, Burton MJ, Leck A (2021). Mycotic keratitis: a global threat from the filamentous fungi. J Fungi (Basel).

[2] Khater MM, Shehab NS, El-Badry AS (2014). Comparison of mycotic keratitis with nonmycotic keratitis: an epidemiological study. J Ophthalmol.

[3] Shen Z, Zhang Y, Li F, Zhang Q (2022). Case report: a rare fungal keratitis caused by Plectosphaerella cucumerina. Ocul Immunol Inflamm.

[4] Kamada R, Monden Y, Uehara K, Yamakawa R, Nishimura K (2012). Rare case of fungal keratitis caused by Plectosporium tabacinum. Clin Ophthalmol.

[5] Sato T, Inaba T, Mori M, Watanabe K, Tomioka K, Hamaya E (2005). Plectosporium blight of pumpkin and ranunculus caused by Plectosporium tabacinum. J Gen Plant Pathol.

[6] Inoue Y, Ohashi Y, Suzuki T (2016). Multicenter prospective observational study of fungal keratitis--current status of patients’ background, clinical findings, treatment and prognosis. Nihon Ganka Gakkai Zasshi.

[7] Yoshida M, Hariya T, Yokokura S (2018). Diagnosing superinfection keratitis with multiplex polymerase chain reaction. J Infect Chemother.

[8] Balne PK, Reddy AK, Kodiganti M, Gorli SR, Garg P (2012). Evaluation of three PCR assays for the detection of fungi in patients with mycotic keratitis. Br J Ophthalmol.

[9] Vengayil S, Panda A, Satpathy G, Nayak N, Ghose S, Patanaik D, Khokhar S (2009). Polymerase chain reaction-guided diagnosis of mycotic keratitis: a prospective evaluation of its efficacy and limitations. Invest Ophthalmol Vis Sci.

[10] Yoshida M, Kiyota N, Maruyama K (2018). Endogenous fusarium endophthalmitis during treatment for acute myeloid leukemia, successfully treated with 25-gauge vitrectomy and antifungal medications. Mycopathologia.

